# Neighbourhood health inequity and the incidence of stroke and transient ischemic attack

**DOI:** 10.1080/02813432.2026.2747501

**Published:** 2026-10-29

**Authors:** Axel C. Carlsson, Yassine Turki, Charlotte Ivarsson, Tessa Schillemans, Boel Brynedal, Ann-Helen Patomella

**Affiliations:** a Division of Family Medicine and Primary Care, Department of Neurobiology, Care Sciences and Society, Karolinska Institutet, Huddinge, Sweden; b Academic Primary Health Care Centre, Region Stockholm, Stockholm, Sweden; c Division of Neurogeriatrics, Department of Neurobiology, Care Sciences and Society, Karolinska Institutet, Solna, Sweden; d Centre for Epidemiology and Community Medicine, Region Stockholm, Stockholm, Sweden; e Department of Global Public Health, Karolinska Institutet, Stockholm, Sweden; f Division of Occupational Therapy, Department of Neurobiology, Care Sciences and Society, Karolinska Institutet, Stockholm, Sweden

**Keywords:** Socioeconomic status, neighbourhood, stroke, TIA, Sweden, sex differences

## Abstract

**Introduction:**

Despite generous welfare systems and equitable built environments previous research has shown clear differences in cardiovascular health between areas with different socioeconomic status (SES) in Sweden. To direct public health initiatives, there is a need for updated data of SES differences in stroke and whether transient ischemic attack (TIA) incidence also differs based on neighbourhood SES.

**Patients and methods:**

Within the Multimorbidity Integrated Registry Across Care Levels in Stockholm cohort (MIRACLE-S), encompassing healthcare visits and diagnoses from all care levels, we followed 1.4 million individuals without a history of stroke or TIA for 12 years (2014–2025). We used Cox and Poisson regression and analysed both the baseline risk prospectively and a time updated analysis in different neighbourhood-SES.

**Results:**

Neighbourhoods with low SES had a higher incidence of stroke. Among both men and women, SES difference in stroke incidence were greatest before age 65, with no significant differences at age 80 or older. For TIA, we observed a higher incidence in high SES neighbourhoods, particularly among men.

**Discussion:**

The higher stroke incidence in low SES neighbourhoods indicates a need for targeted prevention. Low awareness and underdiagnosis may explanation the lower risk of TIA in low SES neighbourhoods, supporting targeted efforts to improve TIA detection, which could in turn help prevent stroke. Effective mitigation likely requires geographically focused medical care and primary prevention in low-SES neighbourhoods.

## Introduction

Globally, stroke is ranked as the second – following ischemic heart disease – leading cause of disability adjusted life years in age groups above 50 [1]. Socioeconomic disparities in health outcomes remain a major public health challenge. Neighbourhood socioeconomic status (SES) refers to the collective social and economic conditions of a geographic area, typically measured using aggregated indicators such as income, education, employment, housing quality, and crime levels [2]. Neighbourhood and individual-level SES are strongly associated with various health outcomes, including cardiovascular events [2,3], which may provide opportunities for prevention.

There are marked differences in the exposure to environmental and behavioural risk factors between neighbourhoods with different SES [4–7]. For example, the highest prevalence of daily smoking in Stockholm County is found in an impoverished city district (13.6%) and the lowest in a high-SES municipality (1.7%) [8]. Notably, a Swedish study on patients with atrial fibrillation showed that persons living in low SES-neighbourhoods had lower rates of prescriptions for stroke preventive medication (Warfarin) compared to persons living in other areas [9]. These statistics indicate a need for targeted preventative interventions for specific geographical areas. An earlier study analysed the risk of stroke in different neighbourhoods in Sweden in individuals below 50 years of age and found that stroke incidence was significantly higher in deprived neighbourhoods [10]. A study on cardiovascular mortality in Norway shows very similar results as the Swedish study [11] and a recent review concludes a persistent association between lower SES and stroke risk [8]. Moreover, in these studies, the neighbourhood risk remained significant after adjustment for individual-level socio-economic factors such as marital status and education level [8,10,11]. The health divide has increased in Sweden since the previous study was undertaken [10], approximately 15 years ago. Moreover, there is a lack of information on the risks in older individuals and regarding transient ischemic attacks (TIA).

Thus, there is need for up to date and enhanced knowledge on the difference in cardiovascular health between neighbourhoods with different SES. Since stroke risk increases with the prevalence of risk factors such as hypertension, diabetes and atrial fibrillation and both these risk factors and stroke itself increase strongly with higher age, it is of interest to investigate stroke in different age groups [12]. Particularly older individuals are of interest as stroke risk is highly age dependent, risk factors may have accumulated and as the SES-trajectories may be increasingly diverse over time. Furthermore, TIA have as far as we know not been studied with regards to neighbourhood-SES. TIA is of interest, as it may, when detected, help clinicians to identify individuals at high risk of stroke. In those identified as having high risk, lifestyle advice and preventive pharmacotherapies may be initiated. Thus, we aimed to study the risk of stroke and TIA in Stockholm County, hypothesizing that risks of stroke are higher in low SES-neighbourhoods and lower in high SES-neighbourhoods. The hypothesis for TIA is more exploratory. We used time-updated analyses over a long period of time (11 years) to account for and portray the risk time in each neighbourhood category and its effects on the risk of stroke and TIA. We also conducted an analysis of a closed cohort of individuals in different neighbourhoods to show their prospective effects on the risk of stroke based on neighbourhood category during a baseline period.

## Methods

### Study population

This study used real-world-data from the Multimorbidity Integrated Registry Across Care Levels in Stockholm cohort (MIRACLE-S) [13], encompassing healthcare visits and registered ICD-10 codes from all levels of care in all adults [14–16]. The baseline period was from January 1, 2010, to December 31, 2013 and the follow-up period started January 1, 2014, until first stroke or TIA event, moving in or out of Stockholm County or to the end of the follow-up, December 31, 2024, whichever occurs first. The inclusion criteria for this study were having visited a primary care centre or hospital in Stockholm County at any time during 2010 to 2013 and being born after 1996 (age ≥18 at the start of the follow-up, 2014). This provided a study population of *n* = 1,697,048. To increase the internal validity of the findings, we then excluded any individuals moving in or out Stockholm County during baseline 2010–2013. Lastly, to avoid an overestimation of disease incidence due to inclusion of prevalent diseases immediately after study initiation (left censoring), we applied a 4-year wash-out period to exclude prevalent cases of stroke and TIA (excluding those with any diagnosis date prior to start of follow-up; January 1, 2014). This resulted in a final study population of *n* = 1,374,610 (Figure 1).

**Figure 1.**
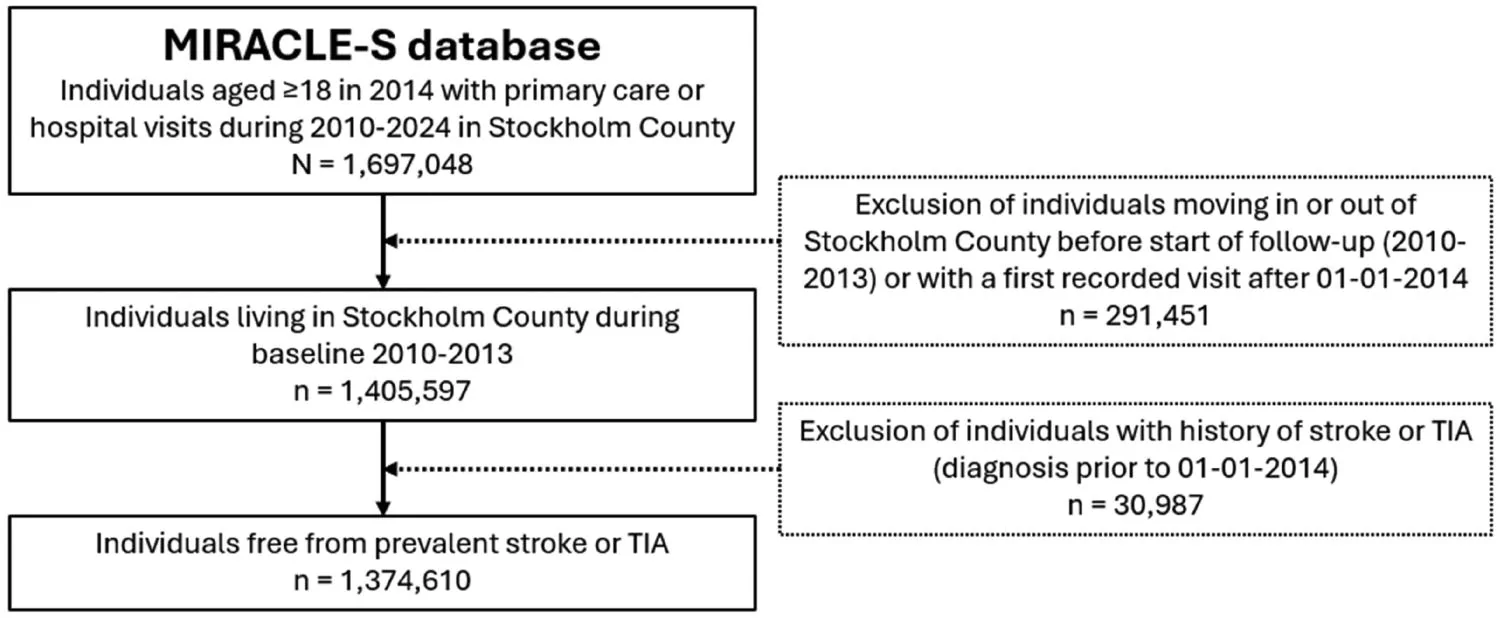
Flow chart with population inclusion numbers.

### Exposure: residential area socioeconomic status

We used residential area as a marker of neighbourhood-SES (categorical: low/moderate/high). We used the mosaic system, Experian’s Mosaic Public Sector 6 (MPS6) © Experian 201,420 [10]. This system was originally developed by the marketing company Experian to classify consumers: Experian, *Mosaic United Kingdom: the consumer classification for the UK*, 2004, Experian Nottingham [10]. It uses over 400 variables to group residential areas that have similar characteristics into broader groups and is used to classify neighbourhoods into low, medium, and high socioeconomic status for research purposes [17,18]. Its use for characterizing health in small area neighbourhoods has been assessed [19]. In the healthcare utilization data from which the present data set was derived the SES-status was updated every sixth month. Thus, in time-updated models, so 6-month intervals from start of follow-up to end of follow-up; 2014 to 2024 was used.

We used 3 categories as for residential exposure: ‘High neighbourhood-SES,’ ‘Medium neighbourhood-SES’ and ‘Low neighbourhood-SES’ and using Medium neighbourhood-SES as the reference category. For the fixed effects model, the neighbourhood-SES category was based on the SES during baseline 2010 to 2013. In the main fixed-effects model, we excluded those who moved between residential SES categories during baseline 2010–2013. In sensitivity analyses for the fixed effect models, we included separate categories for those who moved between neighbourhood-SES categories and additionally investigated those with missing neighbourhood-SES. This provided 6 categories: ‘High neighbourhood-SES,’ ‘High & Medium neighbourhood-SES,’ ‘Medium neighbourhood-SES,’ ‘Low & Medium neighbourhood-SES,’ ‘Low neighbourhood-SES’ and ‘Missing’ neighbourhood-SES, again using Medium neighbourhood-SES as reference category.

### Outcome

We identified patients with stroke using ICD-10 codes for the first event using all strokes combined (I60-I69), ischemic stroke (I63), haemorrhagic strokes (I60, I61, and I62) and Transient Ischemic Attacks (G45).

### Statistical analysis

Follow-up started on January 1, 2014 and continued until the date of first event, emigration from Stockholm County, death, or December 31, 2024, whichever occurred first. Time since start of follow-up was measured in days (January 1, 2014), was used as the underlying time scale.

All models were stratified by sex and age at baseline, grouped into 18–44, 45–64, 65–79, and ≥80 years. Within each sex-age stratum, we estimated hazard ratios (HRs) with 95% confidence intervals (CIs) using time-updated Cox proportional hazards regression. To assess the robustness of results we additionally extracted incidence rate ratios (IRRs) with 95% CIs using Poisson regression. All models were additionally adjusted for baseline age. For the time-updated analyses, we constructed individual event histories split into 6-month intervals from 2014 to 2024. Only the SES-category was allowed to change over time. In each interval, we used a time-updated SES category defined as the latest SES value observed right before the interval starts, carried forward until a change. The first interval was anchored to start of follow-up (January 1, 2014). Each interval contributed start and end dates, exposure category, event (stroke and TIA) status in that interval, and time at risk. The time-updated Poisson regression models yielded incidence rate ratios (IRRs) comparing stroke and TIA rates between neighbourhood-SES categories, again treating SES as a time-updated covariate.

Fixed-effects Cox and Poisson models using baseline neighbourhood-SES were fitted as secondary analyses to assess the impact of summarising neighbourhood-SES during 2010–2013 into a single baseline exposure. In these analyses, we used 6-categories for SES-neighbourhood history collected during baseline (2010 to 2013).

As an additional sensitivity analysis for the combined stroke outcome, we repeated the Cox regression analysis retaining individuals with prior transient ischemic attack before baseline, while still excluding individuals with prior stroke before baseline.

## Results

### Population description

We included *n* = 1,374,610 individuals in the study. Out of those, 1,118,188 lived in the same neighbourhood SES category the entire study duration (Table 1) and 256,422 moved between categories during study duration (Supplemental Table S1). Age distributions were broadly similar across high, medium and low neighbourhood-SES categories (Table 1), while individuals who moved between neighbourhood-SES categories were generally younger (Supplementary Table S1). Overall, just over half of the cohort were women (52.7%).

**Table 1.** Descriptive characteristics of the study population of those living in high, medium or low socioeconomic status neighbourhoods during baseline (never-movers).

Characteristic	High	Medium (Reference)	Low	Movers/Missing	Total
Study population, *n*	348,195 (25.33)	455,044 (33.10)	314,949 (22.91)	256,422 (18.65)	1,374,610
Female age 18–44	66,336 (19.05)	83,657 (18.38)	63,019 (20.01)	97,850 (38.16)	310,862 (22.61)
Female age 45–64	69,036 (19.83)	86,866 (19.09)	55,125 (17.50)	30,076 (11.73)	241,103 (17.54)
Female age 65–79	33,557 (9.64)	46,617 (10.24)	31,450 (9.99)	13,388 (5.22)	125,012 (9.09)
Female age 80+	11,217 (3.22)	14,640 (3.22)	13,296 (4.22)	8,451 (3.30)	47,604 (3.46)
Male age 18–44	64,733 (18.59)	86,210 (18.95)	68,680 (21.81)	64,894 (25.31)	284,517 (20.70)
Male age 45–64	66,848 (19.20)	84,783 (18.63)	52,506 (16.67)	27,270 (10.63)	231,407 (16.83)
Male age 65–79	29,938 (8.60)	43,095 (9.47)	23,962 (7.61)	11,106 (4.33)	108,101 (7.86)
Male age 80+	6,530 (1.88)	9,176 (2.02)	6,911 (2.20)	3,387 (1.32)	26,004 (1.89)
Female sex, *n* (%)	180,146 (51.74)	231,780 (50.94)	162,890 (51.72)	149,765 (58.41)	724,581 (52.71)
Male sex, *n* (%)	168,049 (48.26)	223,264 (49.06)	152,059 (48.28)	106,657 (41.59)	650,029 (47.29)
Total follow-up, person-years	3,354,474	4,347,067	2,933,902	2,436,986	13,072,429
Any stroke (I6x), *n* (%)	12,954 (3.72)	18,232 (4.01)	13,931 (4.42)	7,647 (2.98)	52,764
Ischaemic stroke (I63), *n* (%)	6,827 (1.96)	9,839 (2.16)	7,528 (2.39)	3,974 (1.55)	28,168
Haemorrhagic stroke (I60, I61 and I62), *n* (%)	2,027 (0.58)	2,827 (0.62)	2,089 (0.66)	1,259 (0.49)	8,202
Transient ischaemic attack (G45), *n* (%)	5,478 (1.57)	6,775 (1.49)	3,869 (1.23)	2,559 (1.00)	18,681

Note: Values are *n* (%) unless otherwise specified. Total includes all six SES categories.

### Any stroke

The results showed that incidence rates of any stroke increased with age in both women and men (Figure 2(A)). Residence in a low neighbourhood-SES was associated with a higher risk of any stroke in ages 18–44, 45–64 and 65–79 for both women and men (Figure 3(A) and Table 2; e.g. ages 18–44: men HR 1.40, 95% CI 1.25–1.57; women HR 1.35, 1.19–1.53), while high neighbourhood-SES was inversely associated with any stroke in the same three age groups (Figure 3(A)).

**Figure 2.**
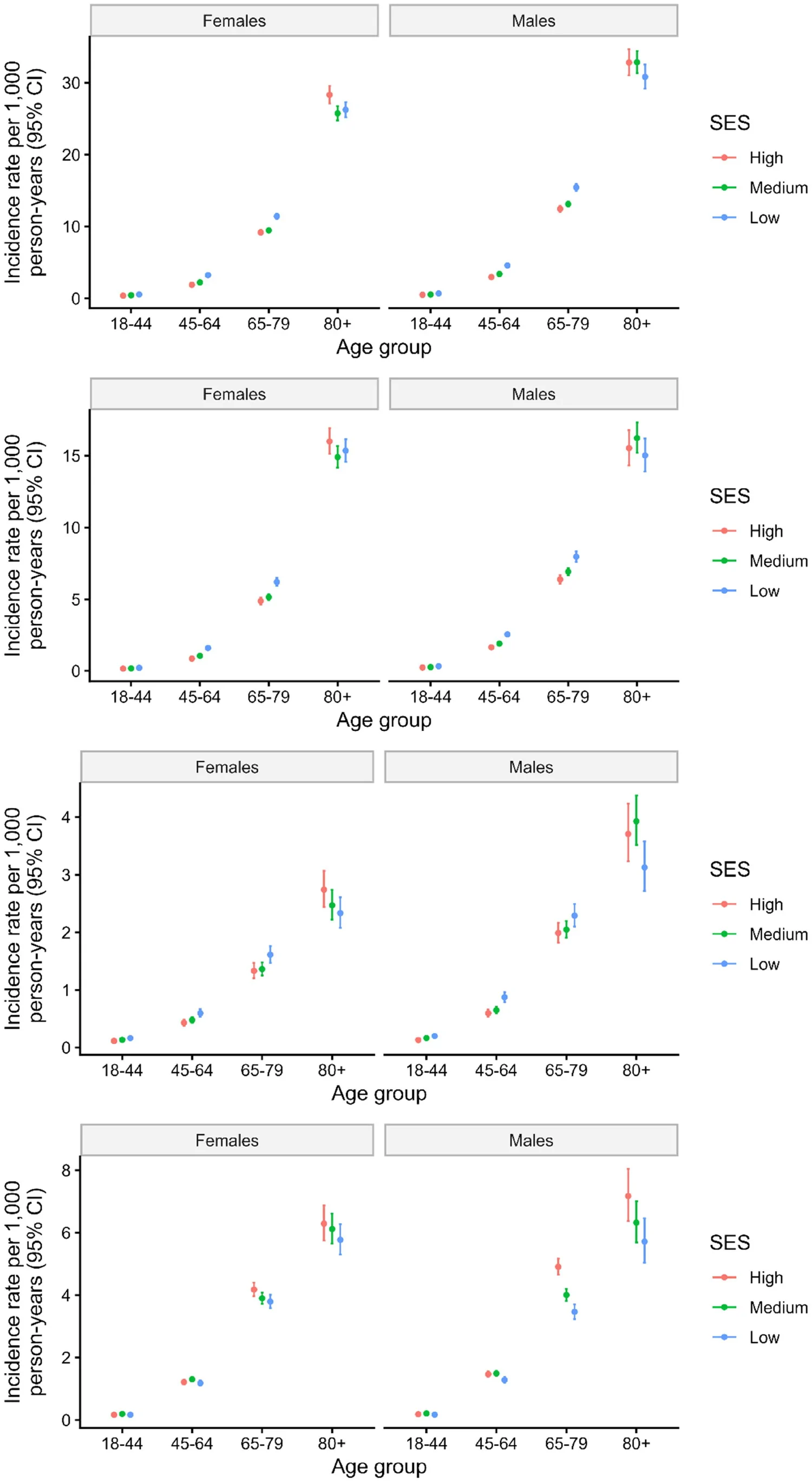
Incidence rates from time-updated models with 95% Poisson confidence intervals for (A) any stroke (B) ischemic stroke (C) haemorrhagic stroke and (D) TIA.

**Figure 3.**
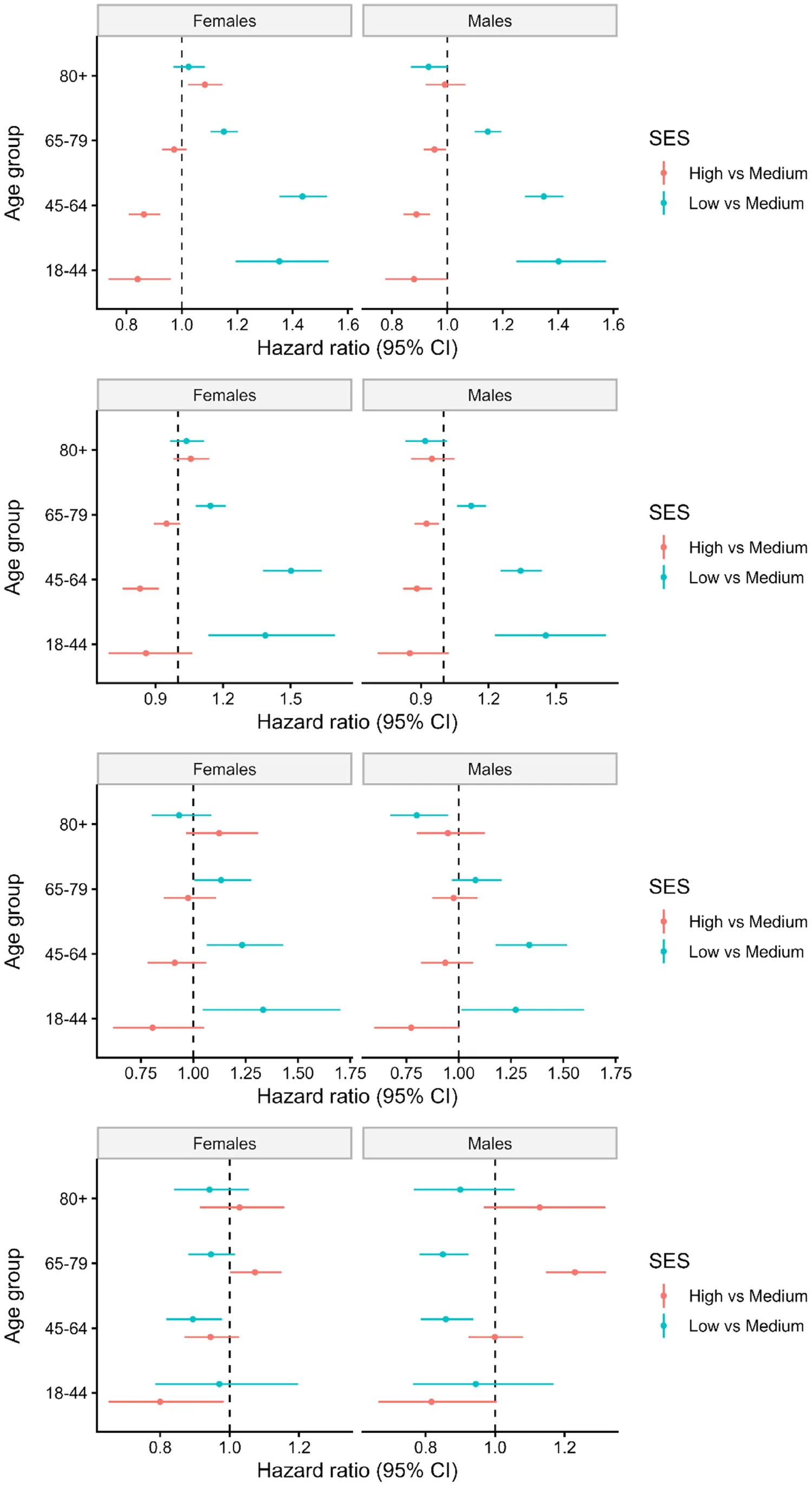
Time-updated Cox regression results for (A) any stroke (B) ischemic stroke (C) haemorrhagic stroke and (D) TIA.

**Table 2.** Time-updated cox regression results for males and females per age category.

	Males		Females	
	*n* events	HR (95% CI)	*n* events	HR (95% CI)
Any stroke				
Age 18–44				
High vs. medium	398	0.88 (0.78–1.00)	349	0.84 (0.74–0.96)
Low vs. medium	547	1.40 (1.25–1.57)	455	1.35 (1.19–1.53)
Age 45–64				
High vs. medium	2,186	0.89 (0.84–0.94)	1,472	0.86 (0.80–0.92)
Low vs. medium	2,629	1.35 (1.28–1.42)	2,027	1.44 (1.35–1.52)
Age 65–79				
High vs. medium	3,549	0.95 (0.91–1.00)	3,098	0.97 (0.93–1.02)
Low vs. medium	3,582	1.15 (1.10–1.20)	3,751	1.15 (1.10–1.20)
Age 80+				
High vs. medium	1,257	0.99 (0.92–1.07)	2,091	1.08 (1.02–1.14)
Low vs. medium	1,302	0.93 (0.87–1.00)	2,395	1.02 (0.97–1.08)
Ischemic stroke				
Age 18–44				
High vs. medium	183	0.85 (0.71–1.02)	136	0.86 (0.69–1.06)
Low vs. medium	254	1.45 (1.23–1.72)	173	1.39 (1.13–1.70)
Age 45–64				
High vs. medium	1,214	0.88 (0.82–0.95)	665	0.83 (0.75–0.92)
Low vs. medium	1,467	1.34 (1.25–1.44)	1,001	1.50 (1.38–1.64)
Age 65–79				
High vs. medium	1,861	0.92 (0.87–0.98)	1,676	0.95 (0.98–1.01)
Low vs. medium	1,899	1.12 (1.06–1.19)	2,080	1.14 (1.08–1.21)
Age 80+				
High vs. medium	622	0.95 (0.86–1.05)	1,223	1.06 (0.98–1.14)
Low vs. medium	658	0.92 (0.83–1.02)	1,443	1.04 (0.97–1.12)
Haemorrhagic stroke				
Age 18–44				
High vs. medium	88	0.77 (0.60–1.00)	86	0.81 (0.62–1.05)
Low vs. medium	138	1.27 (1.01–1.60)	117	1.33 (1.04–1.70)
Age 45–64				
High vs. medium	363	0.94 (0.82–1.07)	272	0.91 (0.78–1.06)
Low vs. medium	432	1.34 (1.18–1.52)	314	1.23 (1.06–1.43)
Age 65–79				
High vs. medium	524	0.98 (0.87–1.09)	398	0.98 (0.86–1.11)
Low vs. medium	535	1.08 (0.97–1.21)	493	1.13 (1.00–1.28)
Age 80+				
High vs. medium	218	0.95 (0.80–1.13)	304	1.12 (0.96–1.31)
Low vs. medium	209	0.80 (0.67–0.95)	308	0.93 (0.80–1.09)
TIA				
Age 18–44				
High vs. medium	144	0.82 (0.66–1.00)	146	0.80 (0.65–0.98)
Low vs. medium	134	0.94 (0.76–1.17)	136	0.97 (0.79–1.20)
Age 45–64				
High vs. medium	1,084	1.00 (0.92–1.08)	943	0.95 (0.87–1.03)
Low vs. medium	742	0.86 (0.79–0.94)	743	0.89 (0.82–0.98)
Age 65–79				
High vs. medium	1,432	1.23 (1.15–1.32)	1,433	1.07 (1.00–1.15)
Low vs. medium	837	0.87 (0.78–0.92)	1,276	0.95 (0.88–1.02)
Age 80+				
High vs. medium	291	1.12 (0.97–1.32)	489	1.03 (0.91–1.16)
Low vs. medium	255	0.90 (0.77–1.06)	551	0.94 (0.84–1.06)

The time-updated Poisson regression models showed very similar patterns (Supplementary Figure S1). The fixed effect models using only baseline SES-neighbourhood confirmed the main findings (Supplementary Figure S2). In the same analysis we found a similar pattern for people that moved between medium and lower neighbourhood-SES categories. However, the people who moved between high and medium categories also showed an increased risk compared to individuals remaining in the medium neighbourhood-SES category (Supplementary Figure S2).

### Ischaemic stroke

Incidence rates of ischaemic stroke also increased with age in both women and men (Figure 2(B)). In the main time-updated Cox models, low neighbourhood-SES was associated with a higher risk of ischaemic stroke in ages 18–44, 45–64 and 65–79 for both sexes, with significant HRs ranging from 1.12 to 1.50. High neighbourhood-SES showed an inverse association with ischaemic stroke in ages 18–44, 45–64 and 65–79 among women, and in ages 45–64 and 65–79 among men (Figure 3(B) and Table 2). In the fixed effect analysis, we observed higher risks among those moving, between medium and low neighbourhood-SES, whereas at older ages those moving between high and medium neighbourhood-SES also had increased risk compared with non-movers in medium neighbourhood-SES (Supplementary Figure S2).

### Haemorrhagic stroke

The incidence of haemorrhagic stroke increased in women and men with age (Figure 2(C)). Low neighbourhood-SES was associated with increased risks of haemorrhagic stroke in the lower age groups, 18–44, 45–64 but not among older persons in the time updated Cox regression models, and the 80+ group of men in low neighbourhood-SES had significantly lower risk of haemorrhagic stroke (Figure 3(C) and Table 2). Poisson models gave similar results (Supplementary Figure S1), and fixed neighbourhood-SES analyses showed the same overall pattern (Supplementary Figure S2).

#### TIA

TIA incidence increased with age in both sexes (Figure 2(D)). In contrast to any stroke and ischaemic stroke, high neighbourhood-SES had the highest risk of TIA: across age and sex groups, the estimates for high and low were generally close to each other, and the confidence intervals often overlapped (Figure 3(D) and Table 2).

Again, Cox and Poisson time-updated models gave very similar results (Supplementary Figure S1), and fixed neighbourhood-SES analyses showed the same overall pattern of weak and inconsistent associations across SES categories (Supplementary Figure S2).

### Sensitivity analysis retaining individuals with TIA

In the additional sensitivity analysis for any stroke, individuals with prior TIA before baseline were retained while individuals with prior stroke before baseline remained excluded. The associations between neighbourhood-SES and any stroke were very similar to the main analysis (data not shown in tables).

## Discussion

### Main findings

In accordance with our hypothesis, there was a higher risk of stroke for individuals living in neighbourhoods with low SES, and a lower risk of stroke among individuals residing in neighbourhoods with high SES, using those in medium SES neighbourhoods as referents. Moreover, we showed that the same risk pattern appeared using two separate strategies, time-updated through follow-up portraying the current risk of living in each type of neighbourhood and baseline fixed effects representing the long-term risk of living in a type of neighbourhood. Both methods did confirm the risk differences in different neighbourhood-SES, one portraying the time in the neighbourhood as the exposure and one the prospective stroke risk. We also found a higher risk of stroke in those who move between neighbourhoods, and interestingly, an attenuation of the differences was seen in the oldest studied group.

For TIA, the findings were less clear, with a tendency towards the opposite pattern, a higher risk in high neighbourhood-SES and a lower risk in neighbourhoods with low neighbourhood-SES. A lower risk of haemorrhagic stroke was also seen in the oldest age-group of men in low neighbourhood-SES.

### Comparisons with other studies

The clear findings concerning stroke are in line with international studies on mortality from stroke [20]; that neighbourhood socioeconomic gradients in mortality from stroke increases substantially and that individuals in the most deprived neighbourhood-SES have a higher stroke mortality. The risk gradients for stroke mortality are also steeper for women than for men. The findings are also in line with a Swedish study of stroke in those under 50 years, where neighbourhood SES was independent of individual level risk factors [10].

We have not been able to find studies describing the risk of TIA in neighbourhoods, making our results novel. One study investigated TIA in Sweden and found that the incidence, unlike other cardiovascular events is not declining [21]. The reason proposed by the authors is that TIA events are more likely to be detected than strokes in those with a favourable lifestyle and an overall low risk. The same reasoning could also be valid for stroke.

### Possible explanations for our findings

There are several possible mechanisms that may explain the higher risk of stroke in low neighbourhood-SES and lower risk in high neighbourhood-SES. It is likely that part of the observed association is mediated trough individual SES profiles, but we could not estimate which proportion due to lack of access to individual level SES factors. The connection between SES and health can go through individual as well as contextual neighbourhood factors. One potential explanation is selection, where poor health at a young age limits the possibility of acquiring a high education, or due to direct social effects as higher education gives the possibility to select occupations that are beneficial to health, and leads to better health literacy. First, higher education and income give more possibilities for a healthy lifestyle and diet, whereas low physical activity as well as a higher use of tobacco is usually observed in low neighbourhood-SES [5]. All these are potential drivers of or risk factors for stroke, such as dyslipidaemia and hypertension [22], as well as stroke itself. Second, a less appealing and less safe built environment i.e. higher crime rate, traffic exposure, and less greenery is also more common in low neighbourhood-SES and may decrease the willingness to go for walks or to spend time outdoors in health-promoting activities [23,24]. Third, low health literacy on when and how to seek care may contribute both to less control of risk factors for stroke as well as to a TIA going unrecognized [25]. It is also possible that strokes may have gone undetected to a higher extent in low neighbourhood-SES, suggesting that the risk of stroke there could be higher than we showed. Fourth, not seeking care when in need was associated with lower age, female sex, being born outside of Sweden, lower educational level, and experiencing discrimination [26]. All these are factors that may be frequent in low-SES neighbourhoods. Finally, there are both higher thresholds to get medical treatment for hypertension as well as stroke preventive medication in those with atrial fibrillation, and these differences have been shown to vary with neighbourhood-SES levels [9], leading to an overall higher stroke risk. Also, individuals with low neighbourhood-SES have been shown to have lower adherence to medication [27], and are also less likely to renew prescriptions due to costs and due to lower health literacy [25].

The risk of stroke in different neighbourhoods was not apparent in the oldest individuals, suggesting that age or health state acts as a leveller. It has been shown previously in Swedish population that the relationship between SES and mortality weakens in older individuals [28]. Possible explanations could be an increasing importance of biological processes for health, survivor bias, or the loss of work-related social gradient in old age.

The fact that TIA may be less common in low neighbourhood-SES may have two explanations. First, individuals are less likely to seek care for milder events such as a TIA, especially in disadvantaged neighbourhoods. Second, milder events such as TIA have been proposed to be more commonly detected in individuals with a healthier lifestyle [21].

### Public health implications

In Sweden, the Health and Medical Services Act mandates that healthcare should be provided on equitable terms, according to the needs of everyone, implying that populations with greater health risks should receive proportionally greater preventive and healthcare resources. Studying differences in stroke incidence between neighbourhoods with different SES is therefore essential to be able to assess whether current healthcare provision focused on cardiovascular disease prevention aligns with this principle of equity. Such knowledge can identify areas with disproportionate disease burden and unmet preventive needs, inform targeted primary and secondary prevention strategies, and support evidence-based allocation of healthcare resources. This means that support and interventions should be focused on low neighbourhood-SES, to bridge the health divide in society. This conclusion would hold true even if observed associations between neighbourhood SES and stroke was completely explained by individual SES levels. The significantly elevated risk of stroke in low neighbourhood-SES presents profound public health implications that extend beyond clinical care, sensing a critical health inequity. This pattern exacerbates social disparities, placing a disproportionate burden of acute treatment, long-term disability, and premature mortality on already disadvantaged communities. Neighbourhood-SES inequity strains healthcare systems through higher volumes of complex, costly cases and increases demand for rehabilitation and social services. The disparity suggests limitations of traditional individual-level prevention, as risk is also shaped by upstream structural determinants: limited access to healthy food, safe environments for physical activity, quality primary care, and chronic exposure to psychosocial stressors [29–31], making geographical placement of prevention interventions logic. The findings suggest that effective mitigation could involve not only medical models but also primary preventative public health strategies, which should be geographically centered in low-SES neighborhoods. In Sweden, the most well-known is the Västerbotten Intervention Programme, a health dialogue intervention within primary healthcare which was initiated to counteract poor health in a vulnerable population in the north of Sweden and shown to impact all-cause and CVD mortality [32]. Further, an RCT found that a lifestyle-based stroke prevention intervention situated in primary healthcare significantly reduced stroke risk at 12 months compared with a control group [33]. However, none of these studies targeted neighborhoods with low SES populations.

### Limitations and strengths

A central strength of this study is the use of high-quality, population-based Swedish healthcare registers, which provide comprehensive coverage of diagnoses and care across primary, ambulatory, and in-hospital settings [14,15]. This ensures near-complete follow-up and robust outcome ascertainment for stroke events within the defined geographic area [34]. Furthermore, the uniform access to care under the Swedish system minimizes confounding from extreme disparities in healthcare access, allowing for a clearer assessment of neighbourhood-level socioeconomic factors. Another strength is the different analyses that we conducted to assure that we both catch the risk of the time spent in the different neighborhoods as well as the prospective risks in those living in each type of neighborhood.

Key limitations must be considered. First, the reliance on physician-coded diagnoses means the true prevalence of stroke risk factors such as hypertension, atrial fibrillation or comorbid conditions could be underestimated if not accurately recorded. Yet, we did have access to diagnoses in all levels of care covering all known diagnoses [35]. Second, our analysis is restricted to individuals who have sought care, potentially missing undiagnosed pathology in the community, which was a probable explanation to the lower risk of TIA that we found and potentially also underestimating the risk of stroke in low neighbourhood-SES. The low risk of TIA in low neighbourhood-SES may thus be due to a biased under detection. Third, we excluded all stroke and TIA events prior to baseline. This methodology could have had different impacts as more TIA could for example have been detected and lead to more exclusions of individuals with high risk from the analysis in high neighbourhood-SES. Yet, one can argue that this is a part of the health disparity that we aim to portray. Yet, we also performed a sensitivity analysis retaining individuals with TIA while excluding all prior stroke events and the results remained essentially unchanged in the main analysis. Fourth, the register data provided only legal sex, limiting our ability to account for gender identity, which may be relevant to psychosocial risk pathways. Fifth, we did not have access to individual SES factors of potential interest such as education level or income. The observed association may thus reflect both contextual characteristics of neighborhoods and the socioeconomic composition of their residents. Sixth, the MOSAIC system for determining the neighbourhood SES is not transparent, but it has been shown to identify small area neighbourhoods with disparities and it has been widely used [18,19]. The study was also limited to Stockholm County, which may also limit the generalizability of other cities and countries and extrapolations of our findings should therefore be performed with caution. However, the gaps in Stockholm are relatively large.

## Conclusions

The higher risks of stroke in those in low neighbourhood-SES call for action. The association between neighbourhood-SES inequity and risk of stroke could be explained by lower awareness of cardiovascular risk factors and treatment or secondary prevention for specific risk factors for stroke. We hypothesize that risk factors for stroke could be opportunistically screened in low neighbourhood-SES and treated locally in primary care centres to lower the overall risk of cardiovascular diseases. The puzzling finding regarding TIA needs to be explored further in future studies. Increased awareness of TIA and stroke symptoms in low neighbourhood-SES could potentially lead to increased detection and lead to better prevention of stroke. The overall findings suggest that effective mitigation could involve not only medical care but also primary preventative public health strategies, which should be geographically centered in low-SES neighborhoods.

## Supplementary Material

Supplementary table and figures 260220.docx

## Data Availability

Data can be made available by contacting halsodata.rst@regionstockholm.se after ethical vetting.
