# Supplementary material for: Neighbourhood health inequity and the incidence of stroke and transient ischemic attack
Source: Scand J Prim Health Care. 2026 Oct 29;44(1):2747501. doi: 10.1080/02813432.2026.2747501 (PMC13721811; doi:10.1080/02813432.2026.2747501)
Supplement: Supplementary file 1 [file IPRI_A_2747501_SM6469.docx]

**Table S1 (Movers).** Descriptive characteristics of the study population among movers (change in neighbourhood SES during the baseline period).

| **Characteristic** | **High and medium** | **Low and medium** | **Missing/Other** | **Movers/Missing total** | **Total population** |
| --- | --- | --- | --- | --- | --- |
| Study population, n | 90,295 (6.57) | 107,694 (7.83) | 58,433 (4.25) | 256,422 (18.65) | 1,374,610 (100.00) |
| Female age 18–44 | 35,756 (39.60) | 39,904 (37.05) | 22,190 (37.98) | 97,850 (38.16) | 310,862 (22.61) |
| Female age 45–64 | 11,400 (12.63) | 12,685 (11.78) | 5,991 (10.25) | 30,076 (11.73) | 241,103 (17.54) |
| Female age 65–79 | 4,315 (4.78) | 6,006 (5.58) | 3,067 (5.25) | 13,388 (5.22) | 125,012 (9.09) |
| Female age 80+ | 2,482 (2.75) | 3,795 (3.52) | 2,174 (3.72) | 8,451 (3.30) | 47,604 (3.46) |
| Male age 18–44 | 21,760 (24.10) | 27,462 (25.50) | 15,672 (26.82) | 64,894 (25.31) | 284,517 (20.70) |
| Male age 45–64 | 9,778 (10.83) | 11,461 (10.64) | 6,031 (10.32) | 27,270 (10.63) | 231,407 (16.83) |
| Male age 65–79 | 3,860 (4.27) | 4,797 (4.45) | 2,449 (4.19) | 11,106 (4.33) | 108,101 (7.86) |
| Male age 80+ | 944 (1.05) | 1,584 (1.47) | 859 (1.47) | 3,387 (1.32) | 26,004 (1.89) |
| Female sex, n (%) | 53,953 (59.75) | 62,390 (57.93) | 33,422 (57.20) | 149,765 (58.41) | 724,581 (52.71) |
| Male sex, n (%) | 36,342 (40.25) | 45,304 (42.07) | 25,011 (42.80) | 106,657 (41.59) | 650,029 (47.29) |
| Any stroke (I6x), n (%) | 2,404 (2.66) | 3,627 (3.37) | 1,616 (2.77) | 7,647 (2.98) | 52,764 (3.84) |
| Ischaemic stroke (I63), n (%) | 1,273 (1.41) | 1,892 (1.76) | 809 (1.38) | 3,974 (1.55) | 28,168 (2.05) |
| Transient ischaemic attack (G45), n (%) | 957 (1.06) | 1,106 (1.03) | 496 (0.85) | 2,559 (1.00) | 18,681 (1.36) |

*Note: Values are n (%) unless otherwise specified. For age/sex rows, percentages are within-column.*

**Figure S1**. Time-varying Poisson regression results for A) any stroke B) ischemic stroke C) TIA

**Figure S2**. Fixed Cox regression results for A) any stroke B) ischemic stroke C) TIA
